# Inhibition of PI3K/AKT/mTOR axis disrupts oxidative stress-mediated survival of melanoma cells

**DOI:** 10.18632/oncotarget.3131

**Published:** 2015-01-29

**Authors:** Heather G. Hambright, Peng Meng, Addanki P. Kumar, Rita Ghosh

**Affiliations:** ^1^ Department of Urology, The University of Texas Health Science Center at San Antonio, San Antonio, Texas, 78229, USA; ^2^ Department of Pharmacology, The University of Texas Health Science Center at San Antonio, San Antonio, Texas, 78229, USA; ^3^ Department of Molecular Medicine, The University of Texas Health Science Center at San Antonio, San Antonio, Texas, 78229, USA; ^4^ Cancer Therapy and Research Center, The University of Texas Health Science Center at San Antonio, San Antonio, Texas, 78229, USA; ^5^ South Texas Veterans Health Care System, San Antonio, Texas, 78229, USA; ^6^ Life Sciences Division, Lawrence Berkley National Laboratory, Berkley, California, 94710, USA

**Keywords:** Reactive oxygen species, oxidative stress, mTORC1, melanoma, Nexrutine^R^

## Abstract

Elevated oxidative stress in cancer cells contributes to hyperactive proliferation and enhanced survival, which can be exploited using agents that increase reactive oxygen species (ROS) beyond a threshold level. Here we show that melanoma cells exhibit an oxidative stress phenotype compared with normal melanocytes, as evidenced by increased total cellular ROS, KEAP1/NRF2 pathway activity, protein damage, and elevated oxidized glutathione. Our overall objective was to test whether augmenting this high oxidative stress level in melanoma cells would inhibit their dependence on oncogenic PI3K/AKT/mTOR-mediated survival. We report that Nexrutine^R^ augmented the constitutively elevated oxidative stress markers in melanoma cells, which was abrogated by N-acetyl cysteine (NAC) pre-treatment. Nexrutine^R^ disrupted growth homeostasis by inhibiting proliferation, survival, and colony formation in melanoma cells without affecting melanocyte cell viability. Increased oxidative stress in melanoma cells inhibited PI3K/AKT/mTOR pathway through disruption of mTORC1 formation and phosphorylation of downstream targets p70S6K, 4EBP1 and rpS6. NAC pre-treatment reversed inhibition of mTORC1 targets, demonstrating a ROS-dependent mechanism. Overall, our results illustrate the importance of disruption of the intrinsically high oxidative stress in melanoma cells to selectively inhibit their survival mediated by PI3K/AKT/mTOR.

## SIGNIFICANCE

Increased incidence of malignant melanoma continues to challenge patients and physicians due to its notorious resistance to therapeutic intervention. Elevated oxidative stress in melanoma tumors allows them to be more aggressive. Therapeutic agents that increase high oxidative stress threshold in cancer cells have shown the potential to selectively target tumor cells. The significance of this work is that Nexrutine^R^; a bark extract augments pre-existing oxidative stress and inhibits the antioxidant response, which culminates in cell death through the suppression of a key survival pathway used by melanoma cells.

## INTRODUCTION

Metastatic melanoma is associated with poor prognosis, disease aggressiveness and resistance to chemotherapeutic strategies [1]. Overall melanoma incidence has steadily risen over the past thirty years [2]. Recently, Lin and colleagues used a model based on incidence, recurrence, death from all causes, SEER data, US census and reports in the published literature to predict the total number of melanoma and advanced melanomas in the US. According to this model, total and advanced melanoma cases will increase by 24.4 and 21% respectively between 2010 and 2015 [3]. Further, although overall cancer-related mortality is reportedly decreasing, this trend does not appear to apply to malignant melanoma. This may in part be due to the lack of a full understanding of the mechanisms involved in melanomagenesis that hinders development of effective therapeutic modalities.

Reactive oxygen species (ROS) are important signaling molecules that are generated as by-products of cellular metabolism and eliminated by antioxidant defense mechanisms in part through the KEAP1/NRF2 pathway [4]. The aberrant accumulation of ROS can result in redox imbalance and subsequent oxidative stress. Evaluation and exploitation of the differential oxidative stress levels and its downstream signaling between normal and cancer cells is emerging as a valuable therapeutic target [5, 6]. Compared with normal cells, cancer cells exhibit increased ROS levels, which is a result of their high metabolic activity [7]. Increased ROS in cancer cells leads to elevated oxidative stress and subsequent activation of growth signaling pathways that favor their survival. Dependence on high ROS renders these cells sensitive to further increases in oxidative stress, especially in melanoma [8–10]. Thus, targeting cancer cells with agents that increase ROS beyond this elevated threshold can lead to selective tumor cell death, with little effect on normal cells [11].

Cancer cell properties like increased proliferative capacity and anchorage-independent growth are mediated in part through PI3K/AKT/mTOR signaling, which controls cell growth through protein synthesis [12]. This signaling is often hyperactive in cancer, including melanoma, and targeting this pathway to reduce cancer cell growth has been a major focus of drug development [13–15]. Given that mTORC1 controls growth and proliferation, its response to ROS would suggest that tumor cells require a chronic level of oxidative stress for their survival and metabolic demands. Since ROS can both activate and inhibit mTORC1 in a context-dependent manner, chronic oxidative stress that occurs in cancer cells is expected to stimulate growth, whereas an overload of ROS results in oxidative damage and mTORC1 inhibition, and therefore inhibits cell growth.

In this study, we examined key differences in redox characteristics between normal melanocytes and melanoma cells and evaluated Nexrutine^R^ as a selective ROS inducer in melanoma. Nexrutine^R^ has been tested in our laboratory for many years for its cancer prevention properties in prostate and pancreas models [16–21]. Its ability to modulate ROS has only recently been realized [22]. We demonstrate that Nexrutine^R^ selectively induces ROS in melanoma cells, resulting in oxidative stress and cell growth inhibition, mediated through PI3K/AKT/mTOR pathway. Moreover, pre-treatment using antioxidant NAC was found to mitigate the effects of Nexrutine^R^ in melanoma cells, including ROS and oxidative stress induction. Our results provide a rationale to test compounds for their ability to preferentially perturb elevated oxidative stress and inhibit survival pathways in melanoma cells as a potential therapeutic strategy.

## RESULTS

### Basal oxidative stress level in melanocytes and melanoma cells

**Figure 1:**
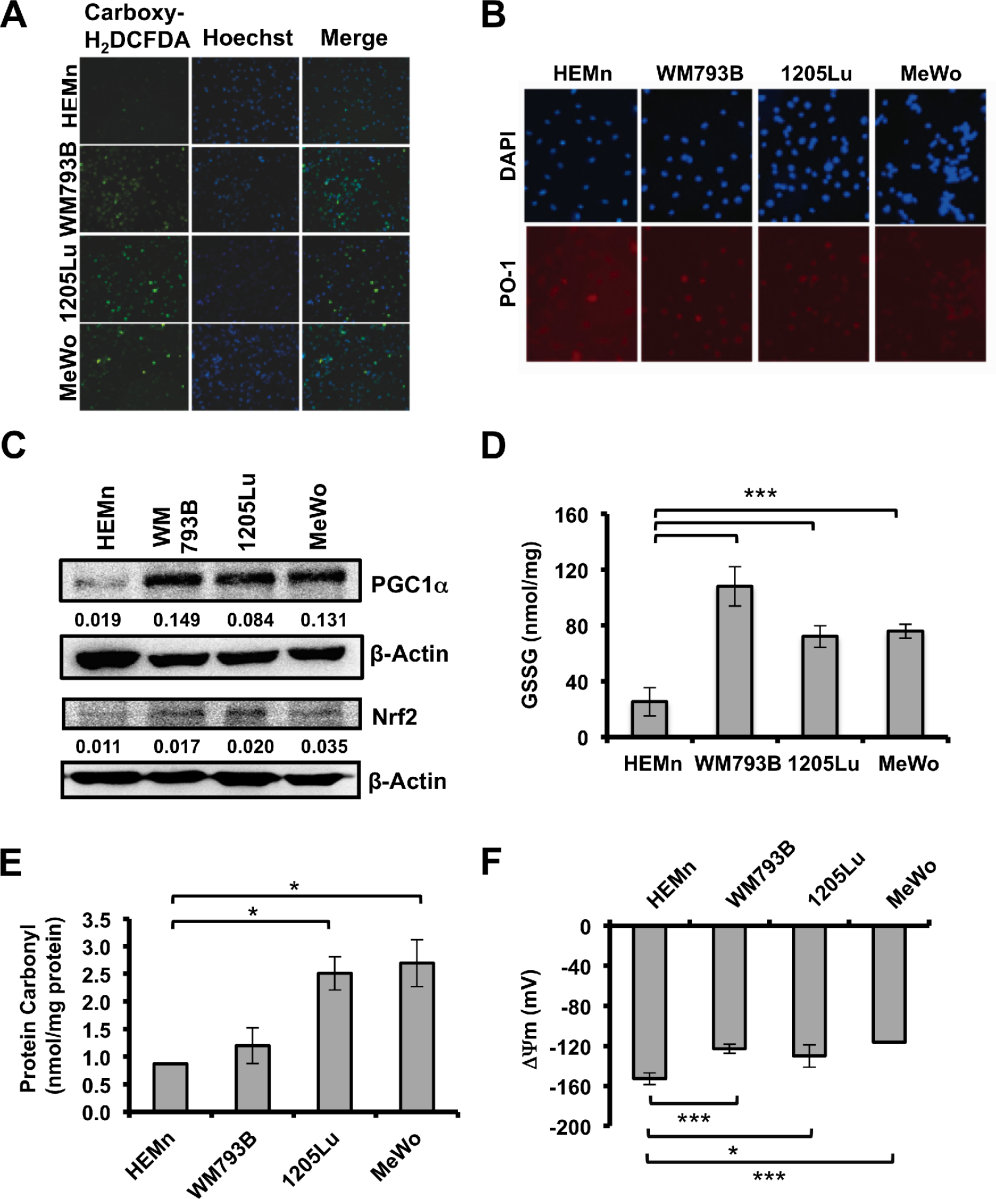
Basal ROS and oxidative stress markers in melanoma cells and melanocytes **(A)** Fluorescent micrographs showing total intracellular ROS by carboxy-H_2_DCFDA, nuclear counterstain by Hoechst, and merged image in melanocytes (HEMn) and melanoma cells (WM793B, 1205Lu, MeWo) at 10X magnification. **(B)** Evaluation of basal H_2_O_2_-specific ROS by Peroxy Orange 1 (PO-1), 20X magnification. **(C)** Basal protein levels of PGC1α and NRF2 by western blotting. Quantification of band densitometry is shown below, relative to β-actin loading control. **(D)** Basal level of oxidized intracellular glutathione (GSSG; nmol/mg protein) determined using luminescence-based assay. **(E)** Intracellular protein carbonylation used as a measure of protein damage, determined by ELISA. **(F)** Mitochondrial membrane potentials (ΔΨ) were determined using Nernst equation derivative. Data are presented as means of three independent experiments. Statistical analysis was performed using Student's *t*-test. Significance values; *indicates *p* ≤ 0.05; and ***indicates *p* ≤ 0.001.

We evaluated multiple oxidative stress markers to determine differences between primary melanocytes and melanoma cells. To evaluate the basal ROS levels in the HEMn melanocyte cells and human malignant melanoma cells WM793B, 1205Lu, and MeWo; we used two independent fluorescent dyes. We observed higher levels of oxidized carboxydichlorofluorescein, a measure of total ROS in melanoma cells compared with normal melanocytes (Figure 1A). Quantification is shown in Supplementary Figure 1A. Further, Peroxy Orange-1 (PO-1), which detects H_2_O_2_-specific ROS, was also higher in melanoma cells compared with HEMn suggesting that part of the total ROS generated is from H_2_O_2_ (Figure 1B). We also analyzed the levels of antioxidant proteins including the master regulator, PGC1α and its target *NRF2*, which constitutes a primary cellular response to chemical and oxidative stress. Protein levels for PGC1α and NRF2 were higher in all 3 melanoma cells compared with HEMn (Figure 1C). Further, we also found that the NRF2 target, NQO1 was significantly elevated in WM793B, and *HMOX1* transcript was elevated in 1205Lu and MeWo compared with HEMn (Suppl. Figure 1B). Subsequently, we determined the overall cell redox status. Under balanced cellular redox conditions, reduced glutathione (GSH) makes up approximately 90% of total glutathione and is constantly converted from the oxidized form (GSSG). Therefore, oxidized glutathione levels are indicative of oxidative stress. We assessed oxidized (GSSG) glutathione levels using a luminescence-based assay. Compared with melanocytes, melanoma cell lines had elevated oxidized glutathione (nmol/mg protein), with the WM793B melanoma cells showing the highest GSSG level (Figure 1D). Overall, oxidized glutathione was significantly elevated (4 to 6 fold) in all melanoma cells compared with melanocytes (Figure 1D). Examination of protein carbonylation, a well-established marker of severe oxidative protein damage showed that all melanoma cells had higher endogenous protein carbonyls compared with melanocytes (Figure 1E). Lastly, we evaluated basal mitochondrial membrane potentials, which reflect intracellular redox homeostasis. In healthy, polarized mitochondria, accumulation of potentiometric dye TMRM can be seen, whereas depolarized mitochondria do not retain the dye and leakage of TMRM is diffused in the cytosol. Confocal imaging of mitochondria allows for quantification of the TMRM fluorescent intensity and used in a Nernst equation derivative, which allows for cellular voltage calculation. We found that all melanoma cell lines had significantly lower basal mitochondrial membrane potentials compared with HEMn cells, indicated by a more positive voltage, which is interpreted as more uncoupled mitochondrial membranes (Figure 1F). Collectively, the data presented in Figure 1 indicate that basal oxidative stress is higher in melanoma cells compared with melanocytes, a feature that might enhance their survival. Therefore, we hypothesized that when melanocytes and melanoma cells are challenged with oxidative stress-inducing agent, the former would exhibit an antioxidant response while the latter would not producing opposite outcomes. To test this hypothesis we used Nexrutine^R^ as the oxidative stress-inducing agent.

### Nexrutine^R^ increases oxidative stress in melanoma cells

**Figure 2:**
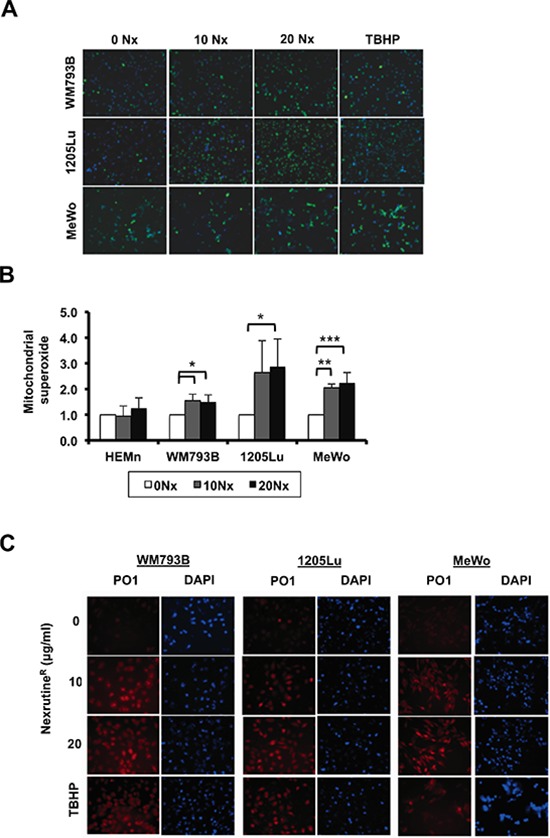
Nexrutine^R^ modulates total and mitochondrial ROS in melanoma cells **(A)** Fluorescent micrographs (10X magnification) merged with Hoechst nuclear stain showing total ROS (by carboxy-H_2_DCFDA) after treatment for 3 h with vehicle control, increasing doses of Nexrutine^R^ (10 and 20 μg/ml), or positive control TBHP. **(B)** Mitochondrial superoxide production, relative to vehicle control in melanocytes (HEMn) and melanoma cells after Nexrutine^R^ (10 and 20 μg/ml; 3 h). **(C)** Induction of H_2_O_2_-specific ROS by Nexrutine^R^ (3 h) evaluated by PO-1 dye in WM793B, 1205Lu, and MeWo, 20X magnification. TBHP (100 μM; 3 h) was used as positive control. Significance values; *indicates *p* ≤ 0.05; **indicates *p* ≤ 0.01 and ***indicates *p* ≤ 0.001.

**Figure 3:**
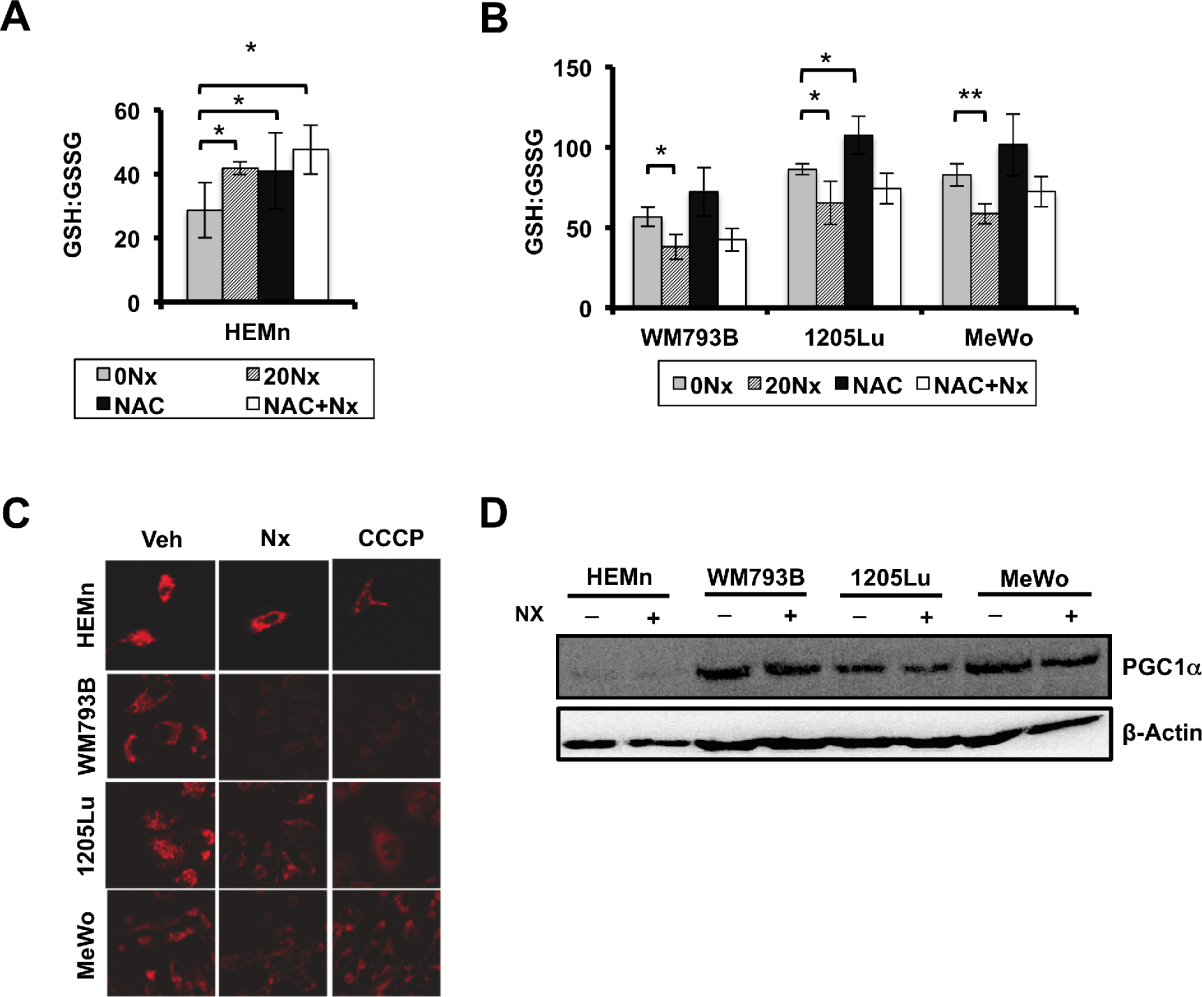
Nexrutine^R^ induces oxidative stress selectively in melanoma cells **(A)** Effect of Nexrutine^R^ treatment (vehicle, 20 μg/ml; 18 h) and NAC pre-treatment (5 mM; 1 h) on ratio of reduced (GSH) to oxidized (GSSG) intracellular glutathione in HEMn melanocytes, determined using luminescence-based assay. **(B)** Effect of Nexrutine^R^ treatment (vehicle, 20 μg/ml; 18 h) and NAC pre-treatment (5 mM; 1 h) on ratio of reduced (GSH) to oxidized (GSSG) intracellular glutathione in melanoma cells, determined using luminescence-based assay. **(C)** Evaluation of mitochondrial membrane potentials by confocal microscopy and incubation with TMRM potentiometric dye after Nexrutine^R^ (20 μg/ml; 3 h), 40X magnification. CCCP was used as a positive control for membrane uncoupling. **(D)** Protein level of PGC1α after Nexrutine^R^ (20 μg/ml; 18 h), evaluated by western blotting. Significance values; *indicates *p* ≤ 0.05; **indicates *p* ≤ 0.01.

Recent work from our laboratory suggests that Nexrutine^R^ modulates ROS in pancreatic cancer cells [22]. As such, we evaluated the potential of Nexrutine^R^ to disrupt the oxidative stress threshold in melanoma cells. Using fluorescence microscopy we found that total ROS levels (carboxydichlorofluorescein) increased in a dose-dependent manner after treatment with Nexrutine^R^ (Figure 2A). Quantification of the imaging data showed an increase in the percentage of ROS-positive cells in all three melanoma cell lines (Suppl. Figure 2A). Validation of ROS levels using flow cytometry showed a higher percentage of ROS-positive cells after 10 and 20 μg/ml Nexrutine^R^ treatment for 3 h compared with control cells (data not shown). To evaluate the source of ROS from Nexrutine^R^ treatment, we determined mitochondrial superoxide production using the Mitosox red fluorescent indicator. We found that Nexrutine^R^ treatment (10, 20 μg/ml; 3 h) resulted in a significant increase in mitochondrial superoxide production in all the melanoma cells that appeared to saturate at 10 μg/ml Nexrutine^R^ (Figure 2B). Interestingly, we did not observe increased superoxide levels in the melanocyte line at 10 μg/ml Nexrutine^R^, and a non-significant increase at 20 μg/ml (Figure 2B). Using PO-1 dye as a probe for H_2_O_2_ production by Nexrutine^R^, we found induction of H_2_O_2_ in all melanoma cells, which is an increment over the basal levels (Figure 2C). Further, we examined the effect of Nexrutine^R^ on intracellular redox balance, using reduced and oxidized glutathione as markers. Strikingly, we found that Nexrutine^R^ treatment resulted in increased glutathione ratio (GSH:GSSG) in melanocytes, which is indicative of reduced glutathione (Figure 3A), which was similar as the effect of NAC treatment. However, Nexrutine^R^ treatment decreased glutathione ratios (GSH:GSSG) in all melanoma cells, indicating increased cellular oxidative stress (Figure 3B). NAC pre-treatment partially abrogated the Nexrutine^R^-modulated glutathione ratio. Evaluation of the effect of Nexrutine^R^ on mitochondrial membrane uncoupling showed that in all melanoma cell lines, Nexrutine^R^ treatment (20 μg/ml; 3 h) resulted in significant decrease in mitochondrial membrane potentials (ΔΨ) compared with vehicle-treated cells (Figure 3C). Melanocytes on the other hand showed no significant effect on mitochondrial membrane potential (Figure 3C). Uncoupling of mitochondrial membranes following treatment are visible as reduced fluorescent intensity due to decreased sequestration of potentiometric dye TMRM in mitochondria, also determined by quantification of membrane potentials (Suppl. Figure 3A). Lastly, we evaluated how Nexrutine^R^ affected the master regulator of oxidative stress; PGC1α. Our results show that Nexrutine^R^ treatment (20 μg/ml; 18 h) decreased PGC1α protein level in all melanoma cell lines unlike the HEMn melanocytes (Figure 3D). Quantification of the protein level is shown in Supplementary Figure 3B. Taken together, these data reveal the differential redox response of melanocyte and melanoma cells to Nexrutine^R^-mediated oxidative stress induction; wherein melanoma cells unlike melanocytes do not activate the antioxidant response leading to increased oxidative stress.

### Nexrutine^R^ changes melanoma cell growth homeostasis

**Figure 4:**
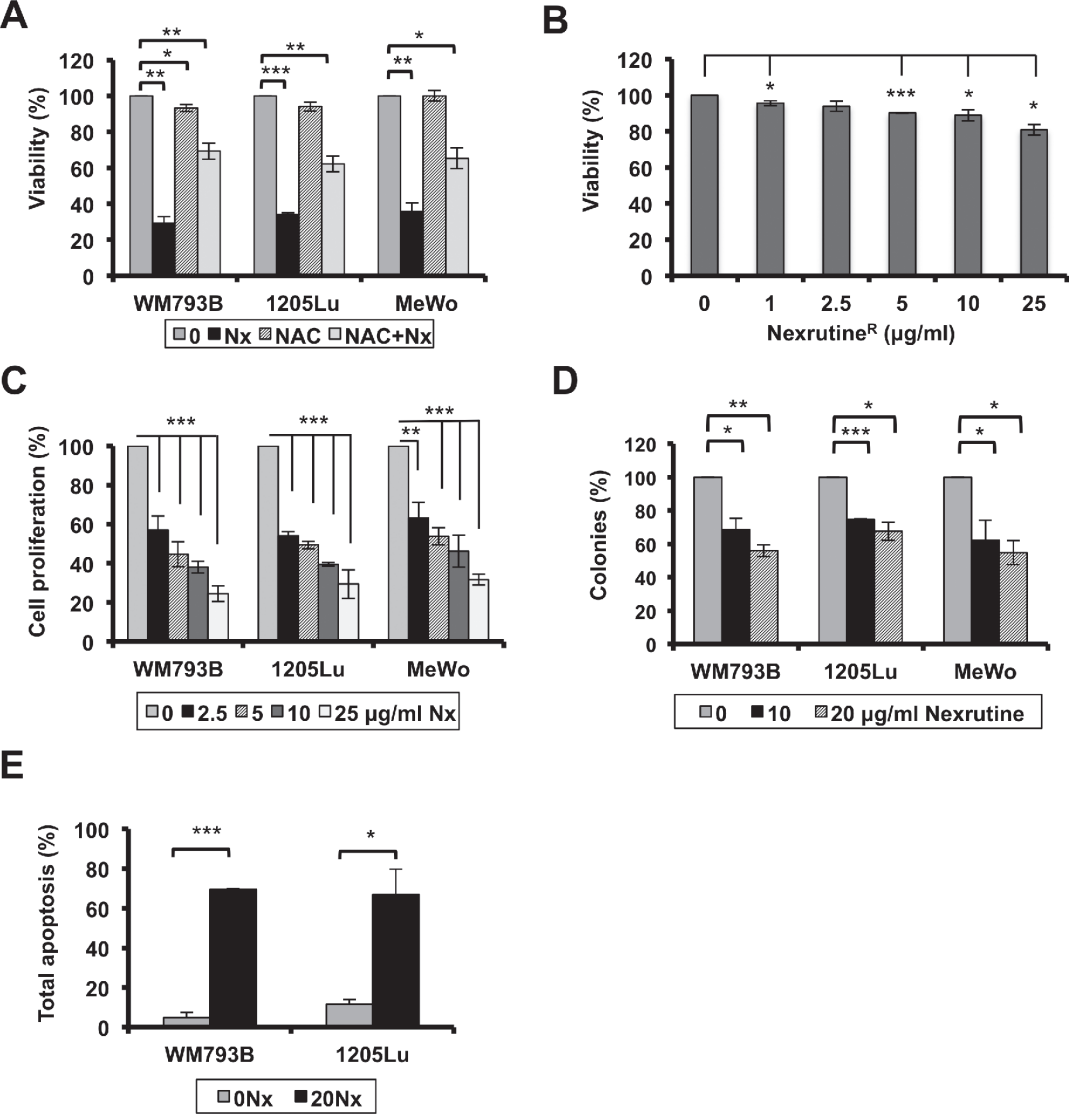
Nexrutine^R^ modulates melanoma cell growth homeostasis **(A)** Effect of Nexrutine^R^ (10 μg/ml; 72 h) on melanoma cell viability (% of control) with and without NAC pre-treatment (5 mM; 1 h) determined by trypan blue exclusion assay. **(B)** Effect of Nexrutine^R^ on HEMn cell viability (live cells, % of control), determined by trypan blue exclusion assay. **(C)** Effect of increasing concentrations of Nexrutine^R^ on cell proliferation, percentage calculated based on vehicle treated controls. **(D)** Nexrutine^R^ inhibits soft-agar colony formation in melanoma cells. Percent colonies based on fluorescence values, normalized to vehicle controls. **(E)** Effect of Nexrutine^R^ (0, 20 μg/ml; 24 h) on melanoma cell apoptosis, determined using Annexin-APC and FACS. Significance values; *indicates *p* ≤ 0.05; **indicates *p* ≤ 0.01 and ***indicates *p* ≤ 0.001.

The next question we wanted to answer was regarding the biological consequence of Nexrutine^R^-mediated increased oxidative stress in melanoma cells. We hypothesized that melanoma cells would succumb to Nexrutine^R^-mediated oxidative stress, while melanocyte cells would not be affected. To examine changes in growth homeostasis, we carried out cellular growth and death assays. We first used Trypan Blue exclusion assay to determine if Nexrutine^R^ treatment changed cell viability. Treatment with 10 μg/ml for 72 h led to more than 60–70% cell death in all three melanoma cell lines, and NAC pre-treatment abrogated Nexrutine^R^-induced cell death significantly (Figure 4A). Nexrutine^R^ decreased viability of HEMn cells by about 20% at the highest dose tested, which was higher than the IC_50_ of melanoma cells (Figure 4B). Examination of HEMn cell morphology changes at the doses of Nexrutine^R^ used in the viability assay showed no signs of apoptosis-associated morphological changes in these cells (data not shown). We found that treatment with increasing doses of Nexrutine^R^ significantly decreased cell proliferation in all three melanoma cell lines in a dose-dependent manner, with IC_50_ of approximately 5 μg/ml for WM793B and 1205Lu, and 10 μg/ml for MeWo cells (Figure 4C). Soft agar assay to determine whether Nexrutine^R^ could inhibit anchorage-independent growth of melanoma cells showed that Nexrutine^R^ significantly inhibited WM793B, 1205Lu, and MeWo colony formation (Figure 4D). We also found a striking inhibition of melanoma cell survival by visual inspection of Nexrutine^R^ treated cells that were re-plated and allowed to grow for 7 days along with vehicle-treated cells (data not shown). To determine whether Nexrutine^R^-mediated decrease in cell viability was due to apoptosis induction we used Annexin V-APC assay and found a significant induction of apoptosis in WM793B and 1205Lu cells (Figure 4E). However, the ability of Nexrutine^R^ to affect cell cycle was minimal, with the most pronounced effect on MeWo cells as indicated by accumulation of cells in G2 phase (Table 1). Taken together, these data suggest that Nexrutine^R^ effectively disrupts melanoma cell growth homeostasis through generation of ROS without affecting the melanocytes tested.

**Table 1:** Effect of Nexrutine^R^ on cell cycle distribution after 24 h treatment (10 μg/ml) in WM793B, 1205Lu, and MeWo cells as determined by PI staining and FACS analysis

***WM793B***	**%G1**	**%G2**	**%S**
0 μg/ml Nx	64.9 ± 4.6	10.0 ± 5.6	25.1 ± 6.0
10 μg/ml Nx	67.7 ± 5.2	12.2 ± 3.5	19.1 ± 3.8
***1205Lu***	**%G1**	**%G2**	**%S**
0 μg/ml Nx	65.7 ± 6.3	14.8 ± 5.2	18.6 ± 1.7
10 μg/ml Nx	59.5 ± 5.8	18.2 ± 4.4	21.6 ± 0.6
***MeWo***	**%G1**	**%G2**	**%S**
0 μg/ml Nx	56.8 ± 3.5	16.3 ± 3.5	23.6 ± 7.2
10 μg/ml Nx	49.2 ± 3.1	29.8 ± 10.7	20.0 ± 6.3

### Induction of oxidative stress inhibits PI3K/AKT/mTOR signaling

**Figure 5:**
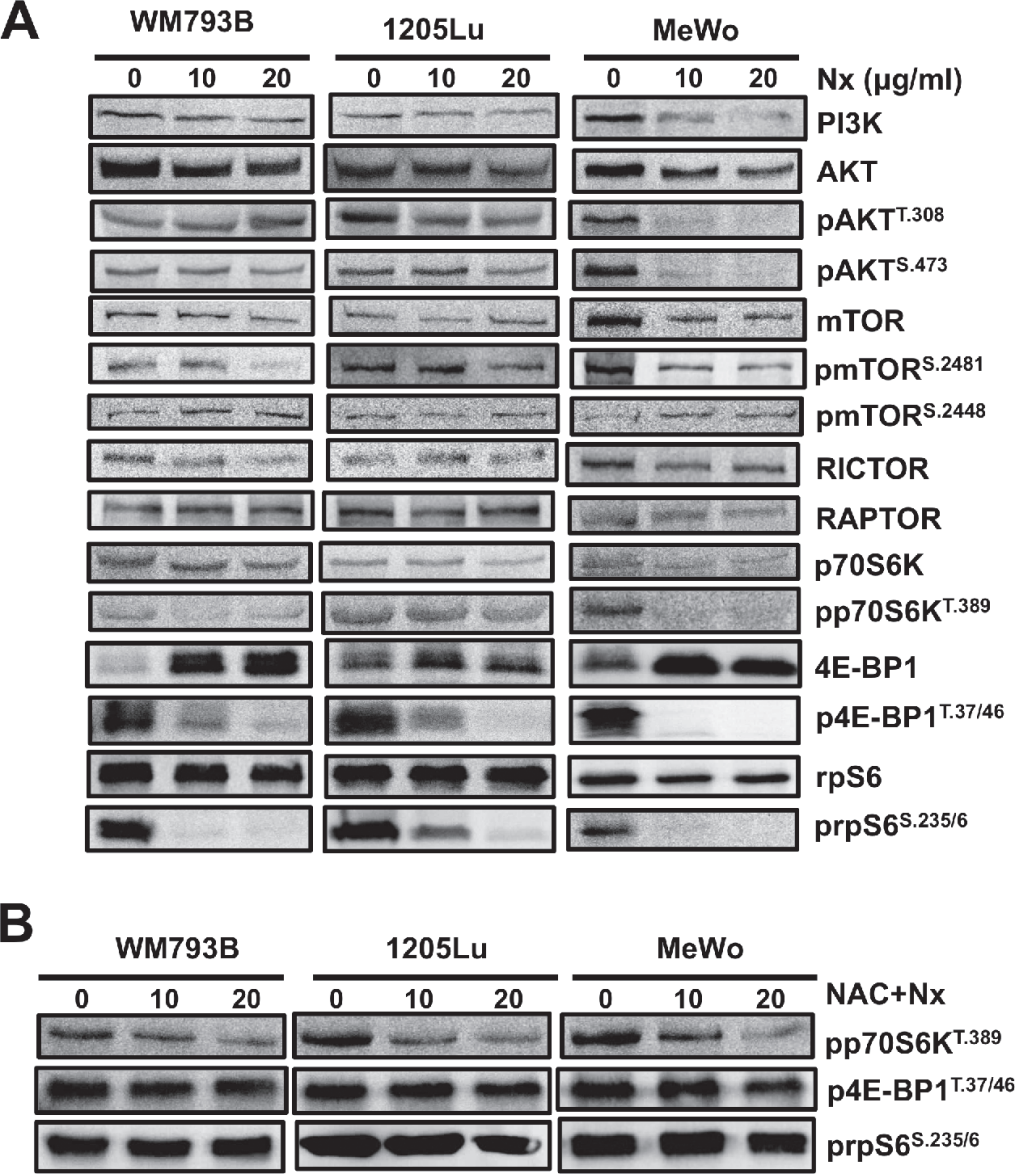
Nexrutine^R^ inhibits PI3K/AKT/mTOR signaling through oxidative stress modulation **(A)** Effect of increasing doses of Nexrutine^R^ treatment for 18 h on total and phospho-protein levels of the PI3K/AKT/mTOR pathway in WM793B, 1205Lu, and MeWo cells, determined by western blotting. β-actin was used as a loading control for each blot. The image is a representative of a minimum of 3 independent experiments. **(B)** Abrogation of inhibitory effect of Nexrutine^R^ on proteins downstream of mTORC1 with NAC pre-treatment (5 mM; 1 h). β-actin was used as a loading control for each blot.

Since we found that Nexrutine^R^ inhibits cell survival and growth, we focused on signaling through the PI3K/AKT/mTOR pathway, which is a major player in growth and survival of cancer cells including melanoma. We found a dose-dependent inhibition of proteins in the PI3K/AKT/mTOR pathway following Nexrutine^R^ treatment (Figure 5A). Treatment inhibited phospho-Akt (Ser^473^) in all the melanoma cells tested, with the most pronounced inhibition in MeWo cells. Additionally, we observed inhibition of p-AKT (Thr^308^) in 1205Lu and MeWo cells, with similar inhibition at both doses of Nexrutine^R^. Interestingly, we observed a slight decrease in total AKT levels with Nexrutine^R^ treatment at both doses tested. This could be due to inhibition of PI3K or a reflection of AKT proteolysis as has been previously reported [23]. Inhibition of phospho-AKT by Nexrutine^R^ resulted in downstream inhibition of mTORC1 target phospho-p70S6K (Thr^389^). The most pronounced effects of Nexrutine^R^ treatment were observed on the inhibition of p-4EBP1 (Thr^37/46^) and p-rpS6 (Ser^235/6^), which are immediate downstream targets of p70S6K. Of note, the low basal level of 4EBP1 and high level of phosphorylated form of the protein may be representative of hyperactive mTORC1 signaling to promote continual cancer cell growth by protein synthesis. Following Nexrutine^R^ treatment, p-mTOR (Ser^2481^) and p-4EBP1 (Thr^37/46^) levels decreased dramatically with a concomitant increase in total 4EBP1 in all the cells. Phosphorylation at serine 2481 is associated with mTOR's intrinsic catalytic activity [24]. We also investigated whether the inhibition of PI3K/AKT/mTOR pathway by Nexrutine^R^ was an effect of increased oxidative stress by pre-treating cells with NAC. NAC pre-treatment decreased Nexrutine^R^-mediated inhibition of phospho-p70S6K, and abrogated Nexrutine^R^-mediated inhibition of phospho-4EBP1, and phospho-rpS6 proteins (Figure 5B). Taken together, these data suggest that Nexrutine^R^ inhibits PI3K/AKT/mTOR signaling in a ROS-dependent manner in melanoma cells.

### Nexrutine^R^-mediated production of reactive oxygen occurs in a rictor and raptor-independent manner

**Figure 6:**
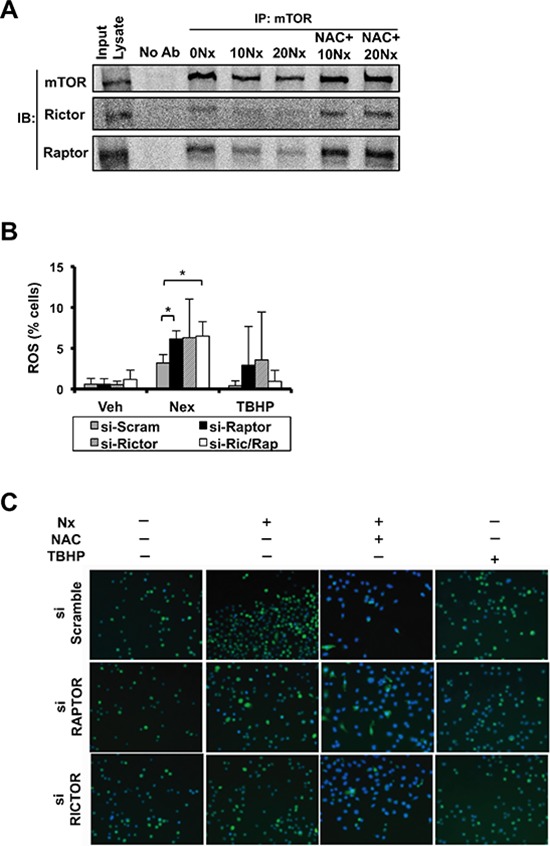
Nexrutine^R^ induces oxidative stress and inhibits mTOR signaling in a RAPTOR/RICTOR-independent manner **(A)** Effect of Nexrutine^R^ (vehicle, 10, 20 μg/ml; 18 h) and NAC pre-treatment (5 mM; 1 h) on disruption of mTOR complex 1 and 2 formation in 1205Lu cells by co-IP using mTOR antibody followed by western blotting with anti-RICTOR, anti-RAPTOR, and anti-mTOR (1:1000) antibodies. 1205Lu whole cell lysate used as input control and pull-down with no antibody as negative control. **(B)** Evaluation of total ROS (% cells) in 1205Lu cells following Nexrutine^R^ treatment (10 μg/ml; 18 h), after transient transfection with siRNA targeting RAPTOR, RICTOR, or both RAPTOR and RICTOR. TBHP is the positive control for ROS induction. **(C)** Evaluation of total ROS by fluorescence microscopy (carboxy-H_2_DCFDA) following Nexrutine^R^ treatment (10 μg/ml; 18 h) and with NAC pre-treatment (5 mM; 1 h) in 1205Lu cells after transient transfection with siRNA targeting RAPTOR and RICTOR. TBHP is the positive control for ROS induction. Representative images of 3 independent experiments are shown. Significance values; *indicates *p* ≤ 0.05.

As discussed above Nexrutine^R^ produced a robust effect on mTORC1 downstream targets such as 4E-BP1, p70S6K and inhibited p-AKT (Ser^473^), which is phosphorylated by mTORC2 complex suggesting that Nexrutine^R^ may inhibit both complexes. RICTOR associates with mTOR upstream of AKT to form the mTORC2 complex and facilitates phosphorylation of AKT exclusively on serine 473. RAPTOR associates with mTOR downstream of Akt and TSC1/2 to facilitate phosphorylation and activation of p70S6K and subsequently, 4EBP1 and rpS6 to control translation and cell size. To determine whether the decreased level of p-AKT (Ser^473^) was mTORC2-dependent, we first evaluated the potential of Nexrutine^R^ to disrupt formation of the mTOR complexes. Using co-immunoprecipitation (mTOR pull down and immunoblot for RAPTOR and RICTOR) we found that Nexrutine^R^ disrupted mTORC1 and mTORC2 complex formation. To our surprise, both RAPTOR and RICTOR were strikingly diminished in 1205Lu cells after Nexrutine^R^ treatment (Figure 6A), but not to the same extent in WM793B or MeWo cells (data not shown). We determined whether Nexrutine^R^-mediated mTORC1 and mTORC2 complex dissociation was ROS-dependent by pre-treatment of cells with NAC and found that Raptor and Rictor protein levels are restored (Figure 6A). To determine whether Nexrutine^R^-mediated ROS production occurs through RICTOR/RAPTOR-dependent manner, 1205Lu cells were transfected with scrambled siRNA or si-*RAPTOR* or si-*RICTOR.* We confirmed knockdown at the mRNA and protein level (Suppl. Figure 4A–4B). Transfected cells were treated with solvent or Nexrutine^R^ and ROS was evaluated using flow cytometry and microscopy. We found that *RICTOR* and *RAPTOR* knockdown had no significant effect on endogenous ROS levels (Figure 6B–6C). However, upon Nexrutine^R^ treatment ROS increased in the scrambled cells as well as RICTOR and RAPTOR knockdown cells suggesting that Nexrutine^R^-mediated ROS production in the 1205Lu cells occurs through RAPTOR- and RICTOR-independent mechanisms (Figure 6B–6C). Pre-treatment with NAC effectively quenched ROS in all these cells (Figure 6C). Overall, the data presented here supports our hypothesis that increasing ROS levels in melanoma cells above the intrinsic threshold of the oxidative stress phenotype can inhibit PI3K/AKT/mTOR-mediated survival and growth.

## DISCUSSION

Cancer cells have a higher metabolic demand to keep up with their rapid proliferation, migration, and invasion needs. Increased metabolism adds to enhanced free radicals production that cells use as signaling molecules to upregulate key signaling pathways including MAPK, VEGF, and PI3K-AKT. To adapt to the high ROS and subsequent oxidative stress, cancer cells may upregulate the KEAP1/NRF2 pathway, a key pathway responsible for sensing and responding to oxidative stress [25]. In the skin, melanocytes have an elevated ROS profile compared with neighboring keratinocytes, which is attributed to melanin synthesis [26, 27]. However, melanoma cells have an even higher level of ROS and elevated antioxidant profile compared to melanocytes [28, 29]. This has led to the hypothesis that targeting addiction to the elevated redox homeostasis may be a therapeutic strategy for malignant melanoma patients.

The observed high levels of ROS and multiple markers of oxidative stress in melanoma cells in our study is in agreement with previous reports that show that cancer cells including melanoma have high constitutive ROS and antioxidant defense pathways to ensure their survival and metabolic demands [30]. We have designated this as the oxidative stress phenotype. Our results also show that the advanced cancer cells have a higher level of oxidative stress, which perhaps is a reflection of their higher metabolic demand compared with primary melanoma cells. More than 50% of malignant melanoma tumors carry *BRAF^V600E^* mutation and about 70% have activated AKT and or mTOR [31, 32]. We did not find significant differences in basal ROS and oxidative stress markers in melanoma cells that represent wild type or mutant BRAF^V600E^, suggesting that the increased ROS may not be associated solely with oncogene-mediated ROS production. Further, the co-existence of increased PGC1α levels and increased oxidative stress in melanoma cells reported here is in agreement with previous findings in melanoma [33].

Nexrutine^R^, obtained from the bark of *Phellodendron amurense*, has been used in our laboratory for a decade to study its anti-prostate cancer activity [16–20]. In a recent phase-I clinical trial, we assessed the safety of Nexrutine^R^ and measured changes in disease burden in patients receiving radiation or prostatectomy and found that Nexrutine^R^ is well tolerated with decrease in PSA in about 81% of patients [34]. Nexrutine^R^ has great potential in other cancer models, including non-melanoma skin and breast cancers [35, 36]. The bark extract has been used in traditional Chinese medicine for hundreds of years. Palmatine, berberine, and jattrorhizine are some of the major components of Nexrutine^R^. Further, the efficacy of Nexrutine^R^ as an anti-cancer agent may be most relevant to cancers with high basal ROS level, as shown in the current study. In very recent work we found Nexrutine^R^ modulated ROS in pancreatic cancer cells [22]. However the utility of Nexrutine^R^ as a ROS-inducer and a disruptor of growth homeostasis in melanoma had not been previously tested. Therefore, we used Nexrutine^R^, as a tool to test whether inducing ROS in an oxidative stress phenotype would disrupt survival of melanoma cells through the PI3K/AKT/mTOR pathway.

Nexrutine^R^ effectively increased ROS and markers of oxidative stress in cells with oxidative stress phenotype. The consequence of these changes was reflected in the disruption of growth, and survival of melanoma cells. The biological effects observed were not significantly changed in the primary melanocyte cells. This suggests that it may be possible to selectively disrupt the elevated redox homeostasis of melanoma cells using Nexrutine^R^ or other agents that can simultaneously disrupt the high redox homeostasis and cell growth and survival pathways. Our observation that Nexrutine^R^ increased mitochondrial superoxide selectively in melanoma cells could be due to the higher levels of the master regulator of antioxidant defense, PGC1α protein. Further experiments will be required to evaluate whether PGC1α is a bonafide molecular target of Nexrutine^R^.

The most pronounced effect of Nexrutine^R^ on the PI3K/AKT/mTOR signaling axis was seen by near complete inhibition of mTORC1 activity as reflected by inhibition of the phospho forms of proteins that are immediately downstream of the mTORC1 complex including 4EBP1, p70S6K, and rpS6, which control translation and cell growth [37]. The profound inhibition of these proteins could account for the observed inhibition of cell proliferation, survival, and colony formation. Inhibitors of AKT/mTOR pathway have been dogged by complications due to activation of AKT by feedback from the TORC2 complex, which phosphorylates AKT at serine 473 to activate it. Nexrutine^R^ inhibited components of the TORC1 and TORC2 complex as well as functional AKT. Since pre-treatment with NAC reversed the inhibition of phosphorylated 4EBP1, p70S6K, and rpS6, it suggests that Nexrutine^R^-mediated inhibition of the PI3K/AKT/mTOR axis is redox-dependent. Of note, prolonged NAC treatment alone has been shown to inhibit mTOR signaling in fibrosarcoma cells by inhibiting ROS-driven mTOR activity [38]. However, the acute, low-dose NAC pre-treatment we employed did not appear to further enhance the mTOR-inhibitory effects seen by NexrutineR. Interplay between ROS and mTOR signaling is complicated, since ROS as a signaling molecule plays both activating and inhibitory roles. Increasing evidence suggests that mTORC1 is a critical ROS mediator [39–41]. Recently, Sorafenib-mediated breast cancer inhibition was shown to be dependent on mTORC1 inhibition and accompanied by mitochondrial membrane depolarization and dramatic ROS increase [42]. Further, work by Yoshida et al. suggests that mTORC1 is a redox-sensitive target with cysteine oxidants destabilizing mTOR-RAPTOR interaction but not mTOR-RICTOR interaction [43]. Given that mTORC1 controls growth and proliferation, its response to ROS in cancer cells would suggest that tumor cells require a chronic level of oxidative stress for their survival and metabolic demands. The effect of ROS on mTORC1 is contextual [44]. Further, our findings that Nexrutine^R^-mediated ROS increase stimulates the KEAP1/NRF2 pathway and inhibits mTOR signaling, could represent an interdependence between NRF2 and mTOR pathways that could be targeted dually by ROS inducers. It is also plausible that the oncogenic function of NRF2 in melanoma cell lines drives high basal ROS and hyperactive mTOR signaling, which has been shown to result from NRF2 mutations [45]. Although the *NRF2* mutational status is not determined in the melanoma lines used, it would be interesting to determine whether mTOR-dependent growth sensitivity of melanoma cells to ROS-inducing agents is related to *NRF2* mutation status. Lastly, adaptation to chronic oxidative stress that occurs in cancer cells would be stimulatory, whereas an acute bolus of ROS might result in oxidative damage, inhibition of mTORC1 and cell survival.

In this regard, redox chemotherapeutics have been used as a therapeutic strategy for melanoma but with limited success. For example, the redox drug Elesclomol showed exquisite selectivity for melanoma cells by very rapid production of mitochondrial ROS-induced apoptosis [46, 47]. However, Elesclomol in combination with paclitaxel did not improve progression-free survival in a randomized, double blind phase III controlled study [48]. Clinical failure of Elesclomol suggests that the next generation of redox chemotherapeutic agents must not only disrupt the high threshold of redox homeostasis but also supress pathways that generate an antioxidant response and allow constitutive upregulation of survival signaling pathways. In this regard, Nexrutine^R^ holds great promise for its ability to disrupt redox homeostasis and target the ROS/mTOR axis with particular implication for melanoma and other cancers. Validation of these *in vitro* findings using Nexrutine^R^ similar to our previous *in vivo* studies is a necessary next step to determine if Nexrutine^R^-mediated one-two punch would be a beneficial agent for melanoma [17, 20].

## METHODS

### Reagents

*N*-acetyl cysteine (NAC) was purchased from Sigma Aldrich (St. Louis, MO). Carboxy-H_2_DCFDA was purchased from Invitrogen (Life Technologies, Grand Island, NY). Peroxy Orange 1 (PO-1) was a kind gift from Dr CJ Chang (Department of Chemistry, UC-Berkeley). Nexrutine^R^ was kindly provided by Next Pharmaceuticals (Salinas, CA). Antibodies were purchased from Cell Signaling Technology (Danvers, MA), Sigma Aldrich (St. Louis, MO), and Santa Cruz Biotechnology (Santa Cruz, CA). Primers for NQO1, HMOX1 and β-ACTIN were designed using Integrated DNA Technologies PrimerQuest software (IDT, Coralville, IA).

### Cell culture

Human melanoma cell lines WM793B, 1205Lu, and MeWo were purchased from the American Type Culture Collection (Manassas, VA) and cultured in MEM media with Earle's salts and L-glutamine supplemented with 0.005 mg/ml insulin, 10% FBS and antibiotics. Normal human epidermal neonatal melanocytes (HEMn) were purchased from Lifeline Cell Technology (Frederick, MD) and cultured as per vendor recommendation. All Nexrutine^R^ treatments were carried out at 10 and 20 μg/ml for 18 h if not otherwise stated.

### Cell proliferation assay

Cells were plated in complete media at a density of 4 × 10^3^ cells per well in a 96-well plate. After 24 h, media was changed to Nexrutine^R^ containing media for 72 h. Proliferation assay was carried out as previously described [19].

### Cell viability, survival, and colony formation assay

Cell viability was evaluated using the trypan blue exclusion method after treatment with Nexrutine^R^ (24 and 48 h). Colony formation was carried out using the CytoSelect 96-well Cell Transformation Assay according to vendor directions (Cell Biolabs, San Diego, CA). Cells were incubated at 37°C at 5% CO_2_ for 7 days after Nexrutine^R^ treatment. Plate was read on SpectraMax M5 plate reader (Molecular Devices, Sunnyvale, CA) using 485/520 nm filters.

### Western blot analysis

Whole-cell extracts were prepared using RIPA buffer (50 mM Tris-HCl, pH 8.0, 150 mM NaCl, 0.1% SDS, 0.5% sodium deoxycholate, 1% Triton X-100) supplemented with fresh protease and phosphatase inhibitors. 100 μg extract was resolved by gel electrophoresis on 8, 10, or 12% SDS-polyacrylamide. Western Lightning Plus ECL chemiluminescent reagent was used as the detection agent. Imaging was performed using GBOX system and protein band quantification using Genetools program (Syngene, Frederick, MD). All proteins were normalized to loading control.

### Co-immunoprecipitation

Whole cell lysates from control and Nexrutine^R^-treated cells were harvested in 0.3% CHAPS lysis buffer with fresh protease and phosphatase inhibitors added. 4 μg of the mTOR antibody (Santa Cruz Biotechnology) were added to 500 μg cleared cellular lysates and incubated on rotary mixer for 90 min at 4°C. 30 μl of 50% slurry of protein G-agarose beads were added per sample and incubated on rotary mixer for 1 h at 4°C. Immunoprecipitated-bead complexes were washed 4 times with CHAPS buffer. Supernates were run on 6% SDS-PAGE gels. Raptor, Rictor and mTOR primary antibodies were diluted to 1:1000 in 5% BSA/TBST and incubated overnight.

### Detection of ROS

Image-iT LIVE Green ROS Detection assay was used to determine ROS according to the manufacturer's recommendation (Molecular Probes, Eugene, OR). Cells were treated with 0, 10, or 20 μg/ml Nexrutine^R^ for 3 h. TBHP (100 μM) was used as a positive control. Carboxy-H_2_DCFDA (25 μM) was added to cells followed by Hoechst nuclear stain (1 μM) for 5 minutes. Cells were imaged on Nikon Eclipse T*i* microscope using DAPI/FITC filter set. Fluorescence data was analysed using Nikon NIS Elements program. To detect H_2_O_2_-specific ROS, cells were incubated with 5 μM Peroxy Orange-1 (PO-1) and imaged using Texas Red filter set. For ROS determination using FACS, cells were washed 1X after carboxy-H_2_DCFDA incubation and processed at the flow cytometry core facility at UTHSCSA using the BD FACS Calibur machine.

### Mitochondrial superoxide determination

Cells were seeded in black tissue culture 96-well plates at a density of 4 × 10^3^ cells/well. After overnight attachment, cells were treated with 0, 10, or 20 μg/ml Nexrutine^R^ for 3 h. Cells were then washed twice in HBSS and incubated with MitoSOX Red Mitochondrial Superoxide Indicator (Invitrogen) in HBSS at a final concentration of 5 μM for 15 minutes at 37°C. Fluorescence was measured using the SpectraMax M5 plate reader using 510/580 nm filters.

### Protein carbonyl determination

OxiSelect Protein Carbonyl ELISA Kit was used according to manufacturer's recommendations (Cell Biolabs, San Diego, CA). Lysates were diluted to final concentration of 10 μg/ml. Carbonylated proteins were detected on the SpectraMax M5 plate reader (Molecular Devices, Sunnyvale, CA) using 450 nm filter.

### Apoptosis assay

Cells were treated with vehicle control or Nexrutine^R^ (20 μg/ml) and harvested by trypsinization after 24 h. Cells were incubated with Annexin-APC and assay carried out according to manufacturer's recommendations (eBioscience, San Diego, CA). Cells were processed at the flow cytometry core facility at UTHSCSA using the BD FACS Calibur machine.

### Glutathione determination

Luminescence-based GSH/GSSG-Glo Assay was used to detect oxidized (GSSG) and total glutathione (Promega, Madison, WI). Cells were pre-treated with NAC (5 mM; 1 h) when necessary, followed by Nexrutine^R^. Assay was carried out according to manufacturer's recommendations and luminescence measured using SpectraMax M5 plate reader.

### RNA interference

Cells were seeded in 6-well plates at 2 × 10^5^ cells/well. After 24 h, cells were transfected with 20 pmol scramble siRNA or siRNA targeting *RAPTOR* or *RICTOR* (Dharmacon, Lafayette, CO).

### Quantitative real-time PCR

1 μg total RNA was reverse transcribed using SuperScript® VILO™ Master Mix (Invitrogen, Life Technologies). 25 ng of reverse transcribed cDNA was used for qPCR amplification in a 7300 Real-time PCR machine using SYBR-Green Real-time PCR Master Mix (Life Technologies, Grand Island, NY). Basal mRNA levels for comparison between cell lines were calculated by ΔCt method.

### Mitochondrial membrane potential determination

Cells were plated at a density of 1 × 10^4^ cells/well in chambered cover glass. Cells were treated with Nexrutine^R^ (20 μg/ml; 3 h). 10 μM carbonyl cyanide 3-chlorophenylhydrazone (CCCP; Life Technologies, Carlsbad, CA) was used as a positive control for mitochondrial membrane uncoupling. DMSO served as negative control and was added at a final concentration of 1%. 30 nM tetramethylrhodamine methyl ester (TMRM; Life Technologies) was added to cells 15–30 minutes before imaging. Cells were imaged on Zeiss 510 NLO confocal microscope using 40X oil immersion lens and HeNe1 laser. Quantification of mitochondrial membrane potentials (ΔΨ) was carried out with ImageJ software (NIH) with Nernst Potential Plugin (Dr J Lechleiter, UTHSCSA).

### Statistical analysis

Data are presented as means ± SD. Statistical analysis was performed using Student's *t*-test. Differences between the control and experimental groups were considered significant at *p* < 0.05; *p* < 0.01 and *p* < 0.001 was considered to be highly significant. Selection of microscopic fields was made at random and representative images were chosen from at least 3 independent experiments. All experiments were repeated at least twice in replicates.

## SUPPLEMENTARY FIGURES


