# Supplementary material for: Inhibition of PI3K/AKT/mTOR axis disrupts oxidative stress-mediated survival of melanoma cells
Source: Oncotarget. 2015 Jan 29;6(9):7195–208. doi: 10.18632/oncotarget.3131 (PMC4466678; doi:10.18632/oncotarget.3131)
Supplement: Supplementary file 1 [file oncotarget-06-7195-s001.pdf]

SUPPLEMENTARY FIGURES

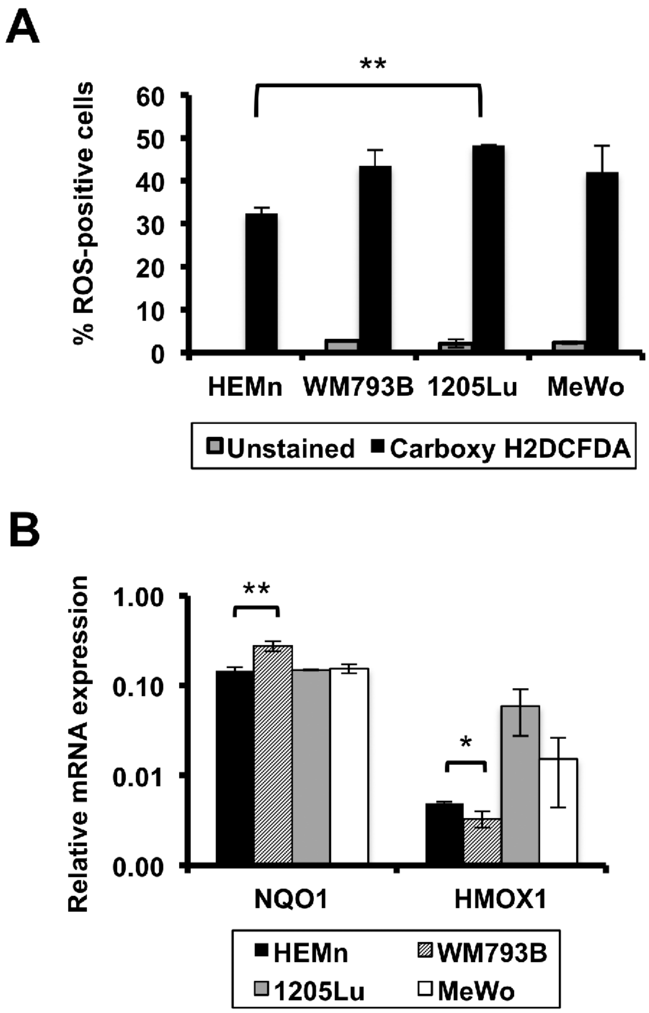

Supplementary Figure 1: (A) Percentage of ROS-positive cells in HEMn melanocytes and WM793B, 1205Lu, and MeWo melanoma cells, determined by flow cytometry after incubation with carboxy-H_2DCFDA. (B) Message level of oxidative stress-response genes *NQO1* and *HMOX1* by qPCR, calculated using $\Delta CT = CT_{\text{target gene}} - CT_{\text{housekeeping gene}}$. Significance was calculated by comparison to HEMn ΔCT values. Significance values; *indicates $p \leq 0.05$; **indicates $p \leq 0.01$.

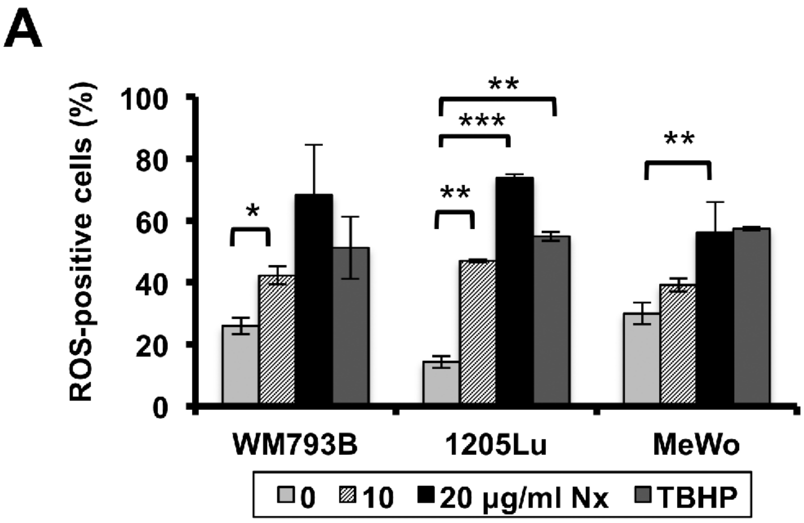

Supplementary Figure 2: (A) Quantification data of Figure 2A to show percentage of ROS-positive cells in field of view after vehicle, Nexrutine[R] (10, 20 µg/ml), or TBHP (for 3 h) using carboxy-H_2DCFDA dye. A minimum of 3 independent experiments was performed. Significance values; *indicates $p \leq 0.05$; **indicates $p \leq 0.01$ and ***indicates $p \leq 0.001$.

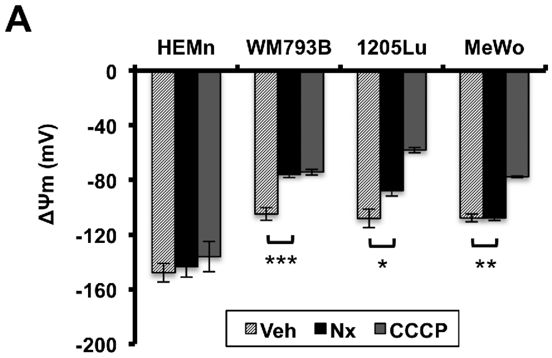

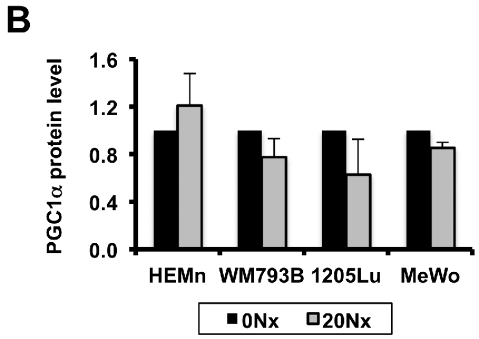

Supplementary Figure 3: (A) Quantification of mitochondrial membrane potential (ΔΨm) changes was calculated from the experimental images of Nexrutine[R] and TMRM shown in figure 3C. A minimum of 50 mitochondria per image and 3 images from independent experiments were used for quantification and statistical analysis. Membrane potentials in millivolts (mV) were calculated using ImageJ Software and a plugin derived from the Nernst Equation. **(B)** Quantification of PGC1α protein level (3 independent experiments) after Nexrutine[R] treatment (20 µg/ml; 18 h). Significance values; *indicates $p \leq 0.05$; **indicates $p \leq 0.01$ and ***indicates $p \leq 0.001$.

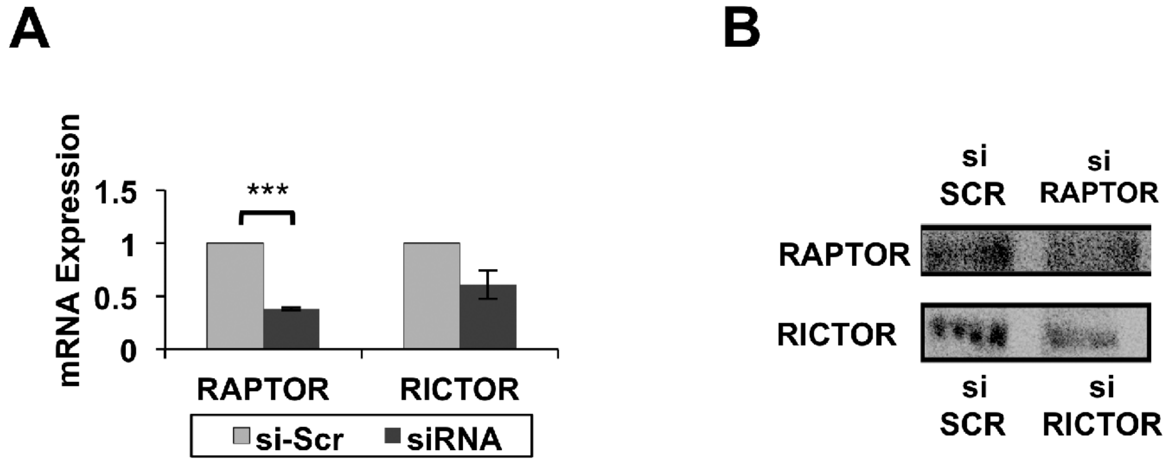

Supplementary Figure 4: (A) Validation of mRNA suppression by qPCR, 48 h after si-*RICTOR* and si-*RAPTOR* transient knockdown in 1205Lu cells. (B) Validation of protein level suppression by western blotting 48 h after si-*RICTOR* and si-*RAPTOR* transient knockdown in 1205Lu cells.
